# Impact of a Standardized Clinical Pathway for Suspected and Confirmed Ileocolic Intussusception

**DOI:** 10.1097/pq9.0000000000000298

**Published:** 2020-05-28

**Authors:** Corinne E. Shubin, Lori E. Rutman, A. Luana Stanescu, Surabhi B. Vora, George T. Drugas, Michael G. Leu, Rebekah A. Burns

**Affiliations:** From the * Department of Pediatrics, Seattle Children’s Hospital, University of Washington School of Medicine, Seattle, Wash; † Department of Radiology, Seattle Children’s Hospital, University of Washington School of Medicine, Seattle, Wash.; ‡ Department of Surgery, Seattle Children’s Hospital, University of Washington School of Medicine, Seattle, Wash; § Department of Biomedical Informatics and Medical Education.

## Abstract

**Introduction:**

Clinical pathways for specific diagnoses may improve patient outcomes, decrease resource utilization, and diminish costs. This study examines the impact of a clinical pathway for emergency department (ED) care of suspected and confirmed pediatric ileocolic intussusception.

**Methods:**

Our multidisciplinary team designed an intussusception clinical pathway and implemented it in a tertiary children’s hospital ED in October 2016. Process measures included the proportion of patients who underwent abdominal radiography, had laboratory studies, received antibiotics, or required admission following reduction of intussusception. The primary outcome measure was the cost per encounter. Balancing measures included unplanned ED visits within 72 hours of discharge. Data analyzed compared 24 months before and 21 months following pathway implementation.

**Results:**

After pathway implementation, the use of abdominal radiography in patients with suspected intussusception decreased from 50% to 12%. In patients with confirmed intussusception, laboratory studies decreased from 58% to 25%, antibiotic use decreased from 100% to 2%, and hospital admissions decreased from 100% to 12%. The average cost per encounter for confirmed intussusception decreased from $6,724 to $2,975. There was a small increase in unplanned returns to the ED within 72 hours but no increase in readmissions after pathway implementation.

**Conclusion:**

Implementation of a standardized ED pathway for the management of suspected and confirmed pediatric ileocolic intussusception is associated with a reduction in abdominal radiographs, improved antibiotic stewardship, reduction in laboratory studies, fewer inpatient admissions, and decreased cost, with no compromise in patient safety.

## Abstract 2

Supplemental Digital Content is available in the text.

## INTRODUCTION

Ileocolic intussusception is one of the most common causes of bowel obstruction in children. The annual incidence in the United States is estimated at 33 to 49 per 100,000 live births.^1^ While other countries have developed national evidence-based guidelines to standardize the evaluation and management of ileocolic intussusception, there are currently no such standards available within the United States.^2^ Consequently, there is considerable practice variation in the evaluation of suspected intussusception, the use of antibiotics surrounding enema reduction, and disposition from the emergency department (ED).^3–5^

Numerous studies have shown improved outcomes with the implementation of clinical pathways for specific pediatric patient populations or diagnoses, including asthma, sepsis, pneumonia, and gastroenteritis.^6–10^

Here we describe the development and implementation of an evidence-based standard pathway for suspected and confirmed intussusception in a pediatric ED. This study aimed to determine the effectiveness of this clinical pathway.

## METHODS

### Context

We conducted the study at a tertiary care, 350-bed academic children’s hospital with a dedicated pediatric ED (50,000 annual visits). Since 2002, our institution has engaged in a hospital-wide initiative to develop standardized pathways for a variety of diagnoses. These are designed with a multidisciplinary team of invested stakeholders and include a systematic review of the literature. Pathways are implemented using flowchart-based algorithms and supported by diagnosis-specific electronic order sets. Process, outcome, and balancing measures are identified a priori and tracked monthly following pathway implementation. Pathways are formally reviewed regularly to ensure they remain consistent with current medical literature and national guidelines. The team makes modifications as necessary using the plan-do-study-act methodology. Our institution has implemented over 70 such pathways with significant improvement in overall clinical care.^11^

### Intervention

**Fig. 1.**
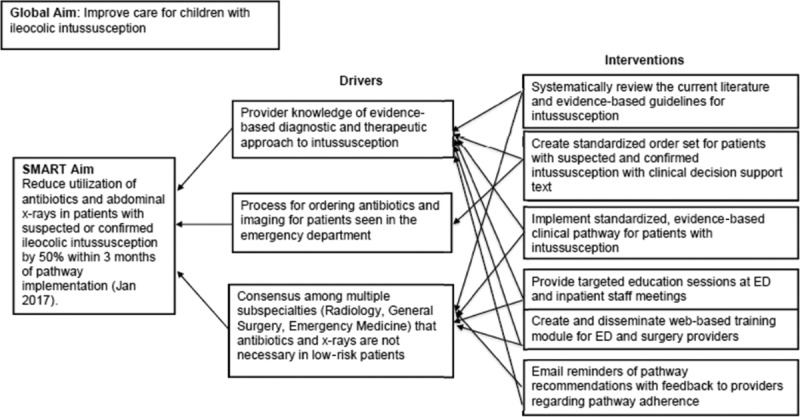
Key driver diagram for intussusception pathway.

We formed a multidisciplinary team of stakeholders to develop a standardized clinical pathway for the evaluation and management of uncomplicated ileocolic intussusception within the ED. This group included physicians (Pediatric Emergency Medicine, Pediatric Radiology, and Pediatric Surgery), a clinical nurse specialist, a pharmacist, an ultrasound technician, and members of the Clinical Effectiveness team, including a consultant, project manager, data analyst, and clinical informatician. The team assessed the current state to identify gaps in standardized care and clinical questions to explore in the literature, including the need for laboratory studies, antibiotics, hospitalization, and the best initial imaging modality. We created a key driver diagram to help inform pathway development and evaluation with SMART (specific, measurable, achievable, realistic/relevant, timely) aims based on current evidence to reduce abdominal x-rays in patients with suspected intussusception and to reduce antibiotic use and laboratory studies in patients with confirmed intussusception (Fig. 1). Key drivers included provider knowledge, the process for ordering imaging studies, and consensus among multiple subspecialties.

A systematic review of the literature was performed using Embase, PubMed, and the Agency for Healthcare Research and Quality National Guideline Clearinghouse. Team members reviewed and rated the quality of the evidence using the GRADE framework to develop a series of recommendations.^12^When evidence was unavailable, the consensus among stakeholders was used. Based on these recommendations, we developed a clinical pathway algorithm (**see Figure 1, Supplemental Digital Content**, which displays intussusception clinical pathway, **http://links.lww.com/PQ9/A183**) and an electronic order-set to guide both the evaluation of suspected intussusception and the management of confirmed intussusception within the ED. The pathway was launched in October 2016. With implementation, we hypothesized that there would be a decrease in abdominal radiographs for the evaluation of intussusception and decreases in the use of antibiotics, laboratory studies, and admission for patients with confirmed intussusception as these were previously standard local practice.

The ED clinical nurse specialist and physician stakeholders led the efforts for pathway implementation. The pathway was discussed at ED, surgery, and radiology meetings before implementation. The ED nurses received specific job aids, and reminders were distributed to physicians at the time of implementation. A web-based training module was required for the ED, radiology, and surgery providers. Finally, we added the pathway to both the internal institutional website housing all clinical standard work pathways and an external website accessible to outside providers.

### Measures

We considered process, outcome, and balancing measures in the analysis. Process measures were chosen to reflect adherence to pathway recommendations. The measures included the proportion of patients with suspected intussusception being evaluated with radiographs and the proportion of patients undergoing therapeutic contrast enemas for the treatment of intussusception who had laboratory studies performed and were given antibiotics or admitted to the hospital after successful reduction. Cost per encounter, as determined by hospital charges, was selected as an outcome measure. Cost data were obtained from hospital administrative records and compared for suspected and confirmed cases (adjusted to 2017 dollars using the medical care component of the Consumer Price Index).^13^ Balancing measures included unplanned returns to the ED within 72 hours of discharge and unplanned return visits within 72 hours with hospital admission. Additionally, we were particularly interested in patients returning to the ED 24 hours following discharge because patients were previously admitted to the hospital for 24 hours following successful reduction. Patients presenting within this window would have previously been under observation. We selected these measures to monitor and identify any unintended consequences of the intussusception pathway, as an increase in ED returns with admission could indicate insufficient care or inappropriate discharge during the initial visit.

### Analysis

We analyzed data for 24 months before and 21 months after the implementation of our pathway in October 2016 (October 2014 to July 2018). Patients were identified retrospectively through our electronic medical record for suspected or confirmed intussusception. Eligible records were identified as suspected intussusception by a provider order at any time during the patient’s ED visit for an abdominal radiograph or ultrasound with “intussusception,” “crampy abdominal pain,” “colic,” or “bloody stool” text in the order detail. Children were excluded from our study population with suspected intussusception if they had an ultrasound specifically performed to evaluate for other indications without stated concern for intussusception, as described above. We identified cases of confirmed intussusception by documentation of a completed fluoroscopic-guided contrast enema performed for intussusception reduction. Exclusion criteria from our study population included signs of complicated intussusception such as hemodynamic instability, peritonitis, bowel perforation, pathologic lead point, or ileo-ileal intussusception. We also excluded patients following an unsuccessful initial contrast enema reduction. Patients with confirmed intussusception were included in data analysis until they no longer met pathway criteria, including patients after failure of reduction on the first attempt.

Descriptive statistics were used to compare demographics of the pre- and post-implementation groups. We used statistical process control to analyze process outcomes and balancing measures. Control charts were created using the QI Charts 2.0 add-on for Microsoft Excel (Process Improvement Products, Austin, Tex.) and analyzed for special cause variation. Data points were aggregated into 2-month groupings. Chi-square tests were used to analyze balancing measures, given the infrequent nature of events.

### Ethical Considerations

Our hospital’s Institutional Review Board approved this study.

## RESULTS

During the study period, 2,164 patients with suspected intussusception met eligibility criteria. There were 179 patients with confirmed ileocolic intussusception, and 145 patients met our pathway criteria and had a successful enema reduction on the first attempt. Demographics of the pre- and post-implementation pathway groups are shown in Table 1.

**Table 1.**
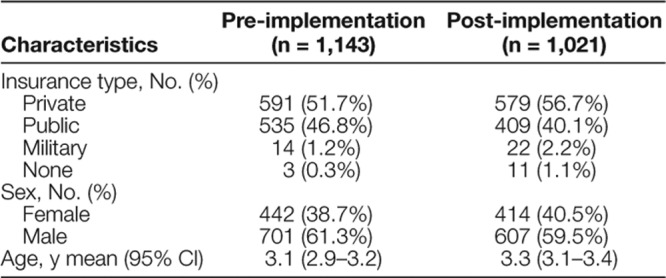
Baseline Characteristics of Subjects Before and After Pathway Implementation

### Process Measures

**Fig. 2.**
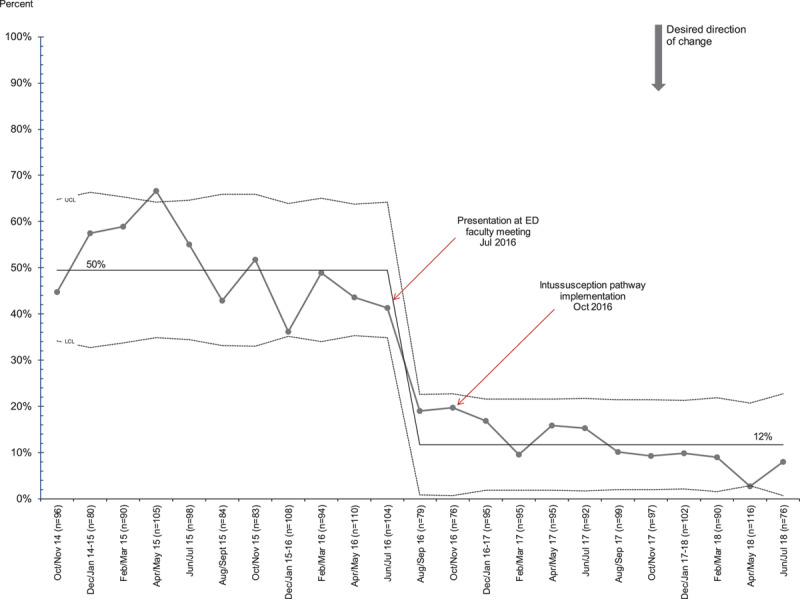
P-chart for the proportion of patients with suspected intussusception evaluated with abdominal radiographs.

**Fig. 3.**
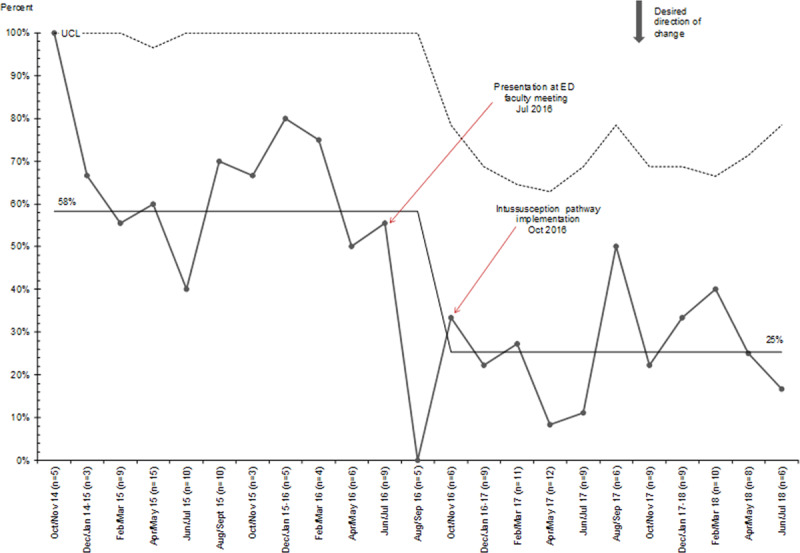
P-chart for the proportion of patients with confirmed intussusception with any laboratory studies done.

**Fig. 4.**
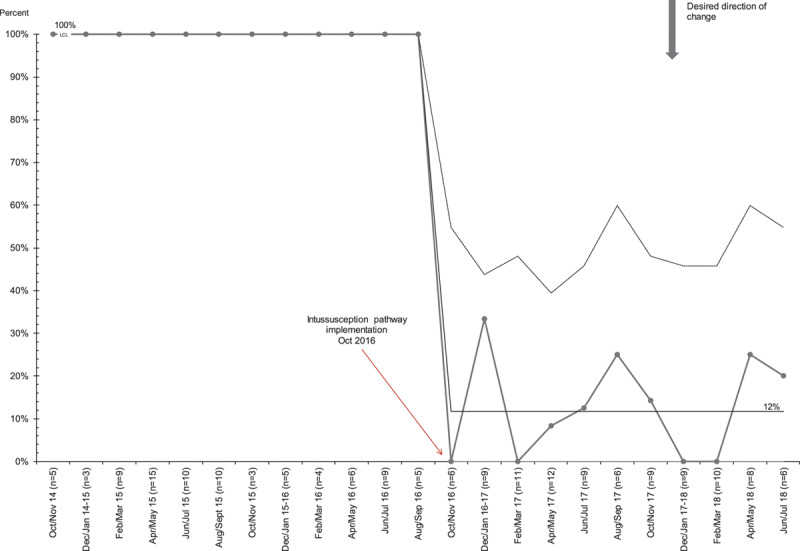
P-chart for the proportion of patients with confirmed intussusception admitted.

In patients with suspected intussusception, we noted special cause variation with a downward shift in the percentage of patients evaluated with abdominal radiographs from 50% to 12% following pathway implementation (Fig. 2). In patients with confirmed intussusception, we noted special cause variation with downward shifts in the proportion of patients with any laboratory studies done from 58% to 25% (Fig. 3). There was also a significant decrease in patients receiving antibiotics from 100% to 2%, with special cause variation evident by a shift of 8 points below the centerline following the intervention. We also found a decrease in the percentage of patients with confirmed intussusception who were admitted to the hospital, from 100% to 12% (Fig. 4). On review of patients admitted after pathway implementation, 2 patients failed the oral challenge in the ED and 3 patients were admitted based on the general surgeons’ recommendations. One patient’s intussusception appeared “hyperdynamic” on ultrasound, and the surgeons were concerned for future recurrence. Two patients had already recurred within 24 hours of a successful enema reduction and prior discharge. Of note, none of these 5 patients experienced a recurrence during their hospitalization. Four additional patients were admitted despite meeting discharge criteria, due to the geographic constraints of our region and the time of night when ED care was completed.

### Outcome Measures

We found a decrease in total direct adjusted costs from an average per patient encounter of $6,724 to $2,975 for patients with confirmed intussusception, with special cause variation noted with a shift of 8 points below the centerline after the intervention. There was no difference in total adjusted costs in patients with negative evaluations before and after implementation.

### Balancing Measures

We found a small increase in unplanned 72-hour return visits to the ED for all reasons in patients with confirmed intussusception on their index visit (7.4% to 16.9%; χ^2^ (1, N = 145) = 3.02, *P* = 0.08). We additionally found a small but insignificant increase in the proportion of patients with confirmed intussusception on their index visit who returned to the ED within 72 hours and were admitted to the hospital (1.5% versus 5.2%, χ^2^ (1, N = 145) = 1.50, *P* = 0.22).

There were 4 children (5.2%) who had recurrent intussusception within 24 hours of their initial reduction and discharge, and they returned to our site for re-evaluation and care. Of these, all 4 had a successful repeat reduction, 2 were discharged home after successful oral trial, and 2 were observed overnight in the hospital. There were no cases of subsequent bowel perforation, severe intra-abdominal infection, or sepsis in children who were discharged home after ED observation.

## DISCUSSION

We used statistical process control analyses to examine the impact following the implementation of a clinical pathway to guide and standardize evidence-based care for patients with suspected or confirmed ileocolic intussusception in a pediatric ED. We assessed how the implementation of this pathway affected the initial imaging modality for suspected intussusception in children, the use of laboratory tests, antibiotic administration, hospitalization, and cost of care for children with confirmed intussusception at a single pediatric tertiary care hospital.

While a common cause of bowel obstruction, children with intussusception often present with nonspecific complaints, such as abdominal pain, fussiness, and emesis. These symptoms can make the diagnosis challenging. Historically, only 21%–32% of North American children with confirmed intussusception have been found to present with the *“*classic triad*”* of vomiting, abdominal pain, and bloody stool.^14,15^ Imaging is, therefore, necessary for diagnosis. Previously, abdominal radiographs were the standard initial diagnostic study at our institution. However, multiple studies have demonstrated that plain abdominal radiographs have a wide range of sensitivity (36%–90%) and specificity (45%–90%), significantly lower than ultrasound which is highly sensitive (98%), and specific (96%–98%) with a high negative predictive value (99.0%–99.7%).^4,16,17^ Ultrasound also lacks the ionizing radiation associated with abdominal radiographs and thus has become the preferred first-line imaging modality for the evaluation of intussusception.^18^

Our results demonstrate that the implementation of a standardized pathway with an emphasis on ultrasound as the preferred imaging modality for patients with suspected intussusception led to a significant decrease in the use of abdominal radiographs. The proportion of patients with suspected intussusception who received a radiograph as part of their ED evaluation decreased from 50% to 12% following implementation of the pathway. While this decline is consistent with our pathway aims, we do not expect radiograph utilization to approach zero in our population. The pathway directs providers to obtain a 2-view abdominal radiograph for patients with ultrasound-confirmed intussusception to evaluate bowel gas and potential free air before enema reduction. The population of patients with suspected intussusception includes those patients who are later positively diagnosed. Thus, we expect that we will continue to obtain radiographs in a small proportion of patients. Although we implemented our pathway in October 2016, we noted a decrease in the use of radiographs beginning in July 2016, corresponding to a presentation to the pediatric emergency department faculty about the new pathway in preparation for go-live.

Historically, many centers, including our own, administered antibiotics routinely to children with intussusception before reduction by contrast enema. This practice was, theoretically, to prevent infectious complications from translocation of bacteria across the gut before or during reduction. However, recent literature has demonstrated similar rates of fever following enema reduction in patients who were and were not treated with antibiotics before the procedure. These studies also showed no difference in time to oral feeds or length of hospital stay between the groups.^19^ Bacteremia immediately before and after reduction appears rare and transient.^20^ Since the implementation of the pathway, the percentage of patients receiving antibiotics at the time of enema reduction decreased from 100% to 2% without any infectious complications.

Before pathway implementation, laboratory tests, including a complete blood count and/or electrolytes, were routinely obtained in patients with confirmed intussusception. These assessments are not valuable in either the diagnosis or management of intussusception as there is no specific finding which allows diagnosis or informs therapeutic interventions in this population.^2^ After the implementation of our pathway, the proportion of patients with confirmed intussusception who had any blood tests decreased from 58% to 25%. Following pathway implementation, some laboratory studies continued to be obtained before the diagnosis of intussusception, likely owing to the nonspecific nature of the symptoms and broad differential being considered before the final diagnosis was made.

Following successful reduction by pneumatic or hydrostatic contrast enema, patients historically were monitored in the hospital for approximately 24 hours to observe for possible recurrence. While recurrent intussusception after initial enema reduction has an incidence of approximately 6%–13%, early recurrence within the first 24 hours and 48 hours has been observed at a rate of 2.2%–3.9% and 2.7–6.6, respectively. This observation suggests that most recurrences will not be identified during a typical hospital admission.^21,22^ Furthermore, recent studies indicate that hemodynamically stable patients who are well appearing may be discharged home with outpatient follow-up and return precautions.^5,21–23^ Discharge after a 4-hour observation period following reduction has not been shown to increase adverse events but was found to reduce costs.^22,23^

Previously, at our institution, all patients with confirmed intussusception were admitted for 24 hours of observation following successful enema reduction. Our new pathway calls for a 2- to 4-hour minimum observation period in the ED following an enema reduction. If the patient tolerates an oral challenge, has a normal abdominal exam, and has reliable access to medical care, they may be discharged home. We found that after pathway implementation, the proportion of patients admitted to the hospital following successful enema reduction dropped dramatically. Patients who were admitted had various reasons such as recent recurrence and living far from our center.

We found that following the implementation of our pathway, the average cost per patient encounter for confirmed intussusception decreased by $3,749. The majority of the savings ultimately reflects that of an overnight hospitalization. However, there is likely some cost savings in the decreased use of intravenous antibiotics and laboratory studies as well. During this study period, on average, there were approximately 40 patients per year who met the criteria for discharge home after reduction and ED observation. Thus, the implementation of this pathway has resulted in a decrease in health care spending of almost $150,000 per year at our institution, in addition to a decrease in resource utilization. Although there was a slight increase in return visits within 72 hours, the average cost of admission for a patient after a successful intussusception reduction was $6,728. In contrast, the cost of a negative ED workup and discharge home for a patient with suspected intussusception was $1,199. Thus, a repeat visit and discharge home following a successful reduction were still less costly than an overnight admission.

The success of our intervention highlights the involvement and buy-in from multiple subspecialties caring for children with intussusception. Local standards of practice before pathway implementation included administering antibiotics to and admitting all patients with confirmed intussusception, as well as obtaining x-rays as the initial imaging modality in patients with suspected intussusception. We found that the use of x-rays initially decreased following provider education at an ED attending meeting. However, the reduced use of antibiotics and admissions did not occur until the pathway was formally implemented, likely reflecting standard practices of the general surgeons before and after implementation.

This study has several limitations. Given the relative rarity of intussusception, we had a small sample size of patients with a confirmed diagnosis. Approximately 8.3% of children presenting with suspicious symptoms were positive on imaging. While we attempted to capture all cases meeting inclusion criteria for our 2 groups of patients before and after implementation of the standard clinical pathway, there is a potential for misclassification given that radiographs are sometimes used as a first imaging modality in children with abdominal pain of unclear etiology early in their clinical evaluation. Additionally, there is the potential for patients being inappropriately included or excluded from our study population, given the necessity to rely on provider comments in the radiology order. However, we expect this misclassification to be similar in both pre- and post-implementation cohorts.

Furthermore, we may not have fully captured patients who had recurrent intussusception or complications after discharge if they presented to other institutions. However, given that our site is the primary children’s hospital for the state, we feel that this is unlikely as these patients would likely have been re-referred to our ED. Finally, our single-center experience reflects our internal culture of high use and buy-in of clinical pathways, which may not be generalizable.

## CONCLUSIONS

Implementation of an evidence-based clinical pathway resulted in decreased use of abdominal radiographs in patients with suspected ileocolic intussusception and reduced use of laboratory studies, antibiotics, and hospital admissions, leading to a reduction in total costs of care for patients with confirmed intussusception. Our results suggest that a standardized clinical pathway for intussusception can positively impact the delivery of care for this population and decrease cost and healthcare resource utilization.

## DISCLOSURE

The authors have no financial interest to declare in relation to the content of this article.

## Supplementary Material


