# Supplementary figures and images for: Impact of a Standardized Clinical Pathway for Suspected and Confirmed Ileocolic Intussusception
Source: Pediatr Qual Saf. 2020 May 28;5(3):e298. doi: 10.1097/pq9.0000000000000298 (PMC7297403; doi:10.1097/pq9.0000000000000298)

**Supplemental figure 1: Intussusception clinical pathway: diagnosis phase**

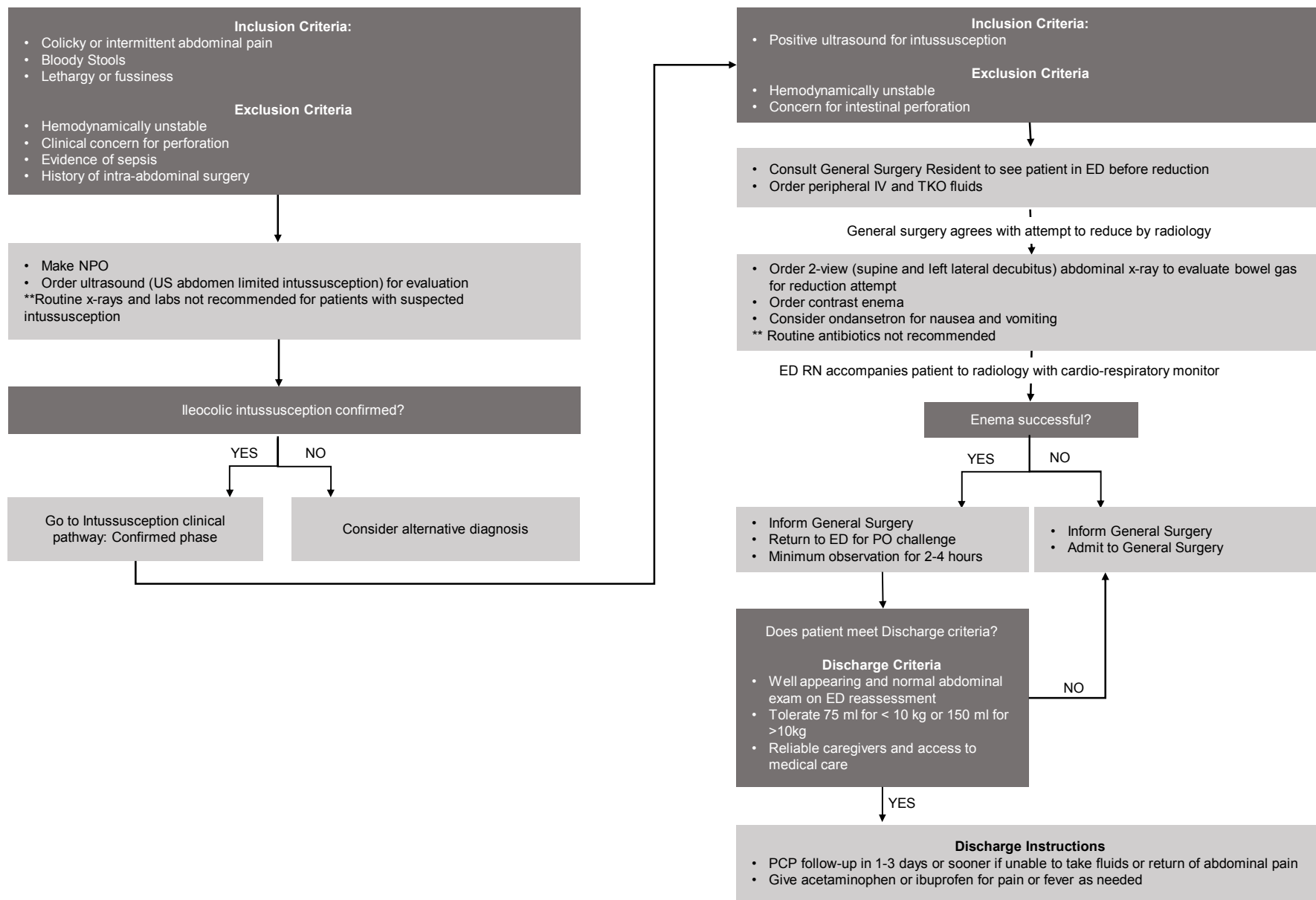

Supplement: Supplementary file 1 [file pqs-5-e298-s001.pdf]
